# Neuron-specific enolase at admission as a predictor for stroke volume, severity and outcome in ischemic stroke patients: a prognostic biomarker review

**DOI:** 10.1038/s41598-024-53080-6

**Published:** 2024-02-01

**Authors:** Matheus Menão Mochetti, Estêvão Garcia Porello Silva, Adriana Aparecida Feltrin Correa, Marcela Rocha Cabette, Iago Navas Perissinotti, Lucas Oliveira Junqueira E Silva, Adriano de Souza Pessoa, Rodrigo Cardoso de Oliveira, Luiz Fernando Ferraz da Silva, Heraldo Possolo de Souza, Júlio César Garcia de Alencar

**Affiliations:** 1 Curso de Medicina, Faculdade de Odontologia de Bauru, Universidade de São Paulo, Bauru, Brazil; 2 Hospital de Base de Bauru, Bauru, Brazil; 3 Hospital das Clínicas da Faculdade de Medicina, Universidade de São Paulo, São Paulo, Brazil; 4 Hospital de Clínicas de Porto Alegre, Universidade Federal do Rio Grande do Sul, Porto Alegre, Brazil; 5 Departamento de Ciências Biológicas, Faculdade de Odontologia de Bauru, Universidade de São Paulo, Bauru, Brazil; 6 Disciplina de Emergências Clínicas, Faculdade de Medicina, Universidade de São Paulo, São Paulo, Brazil

**Keywords:** Stroke, Prognostic markers

## Abstract

An ideal blood biomarker for stroke should provide reliable results, enable fast diagnosis, and be readily accessible for practical use. Neuron-specific enolase (NSE), an enzyme released after neuronal damage, has been studied as a marker for brain injury, including cerebral infarction. However, different methodologies and limited sample sizes have restricted the applicability of any potential findings. This work aims to determine whether NSE levels at Emergency Department (ED) admission correlate with stroke severity, infarcted brain volume, functional outcome, and/or death rates. A systematic literature review was performed using PubMed, Embase, and Scopus databases. Each reviewer independently assessed all published studies identified as potentially relevant. All relevant original observational studies (cohort, case–control, and cross-sectional studies) were included. Eleven studies (1398 patients) met the inclusion criteria. Among these, six studies reported a significant correlation between NSE levels and stroke severity, while only one found no association. Four studies indicated a positive relationship between infarcted brain volume assessed by imaging and NSE levels, in contrast to the findings of only one study. Four studies identified an association related to functional outcome and death rates, while three others did not reach statistical significance in their findings. These data highlight that NSE levels at ED admissions proved to be a promising tool for predicting the outcome of ischemic stroke patients in most studies. However, they presented high discrepancies and low robustness. Therefore, further research is necessary to establish and define the role of NSE in clinical practice.

## Introduction

Ischemic stroke is a major global health issue, ranking as the second leading cause of death and the primary cause of adult disability worldwide^1,2^. Given the broad range of possible outcomes for patients—which can vary from complete recovery to death—and the availability of various treatment options, identifying prognostic factors that can quickly and accurately predict clinical outcomes is crucial^3^.

In this context, the potential benefits of identifying biomarkers involved in the various pathophysiological processes of neurocritical patients have emerged. Among these, S-100B protein, Myelin-basic protein (MBP), and Neuron-specific enolase (NSE) stand out, and the NSE has currently demonstrated promising results in the literature^4–7^. This protein is in the cytoplasm of neurons and plays a role in energy metabolism^8^. As NSE has significant use in several neurological disorders, such as head injury, intracerebral hemorrhage, cardiac arrest, anoxic encephalopathy, encephalitis, and status epilepticus^4^, recent studies have investigated its relationship with ischemic stroke and its characteristics, such as infarcted brain volume, the severity of clinical manifestations, and short- and long-term prognosis^9–12^.

Despite these efforts, current research on the effectiveness of NSE as a blood biomarker for acute ischemic stroke has not provided conclusive results, and its impact on the outcomes of these patients remains poorly understood^9,13^. Moreover, the limited number of patients included in these studies has restricted the applicability of any potential findings^14^.

## Methods

### Study design

This is a systematic literature review that evaluated NSE as a predictor for outcomes in acute ischemic stroke. It followed the Preferred Reporting Items for Systematic Reviews and Meta-Analyses (PRISMA) guidelines.

### Eligibility criteria

We included original observational studies (cohort, case–control, and cross-sectional studies) that evaluated plasma NSE levels at admission to the Emergency Department (ED) among ischemic stroke patients. There was no restriction in terms of language or year of publication. Case reports, narrative reviews, and opinion articles were excluded.

We considered studies with quantitative or qualitative analysis of one or more of the following outcomes: (a) stroke severity (assessed by the National Institute of Health Stroke Scale (NIHSS) and/or Glasgow Coma Scale (GCS) at admission), (b) infarcted brain volume (calculated by Computerized Tomography (CT) and/or Magnetic Resonance Imaging (MRI)), (c) functional outcome (assessed by the modified Rankin Scale (mRS) at discharge and/or in the early days after stroke), and (d) death rates (at discharge and/or in the early days after stroke).

### Search strategy

A systematic literature review strategy was developed and performed by a medical doctor with inputs from the study investigators. This strategy combined keywords and standardized index terms related to NSE levels and ischemic stroke outcomes. It included the search terms Stroke OR Cerebrovascular Accident OR Brain Vascular Accident AND Neuron-Specific Enolase OR Neuron Specific Enolase OR NSE. The initial search occurred in February 2023 on PubMed (507), Embase (667), and Scopus (489) databases.

### Study selection and data extraction

In the first phase, two investigators independently screened all titles and abstracts for eligibility. In the second phase, all studies considered potentially relevant were fully read and independently assessed for eligibility. The investigators were not blinded to the authors, journals, or study results.

Relevant data were independently extracted from all studies using a standardized and predefined extraction form. The extracted data included author, journal, year, country, number of patients, outcome measurement, and outcomes. Unadjusted and adjusted effect estimates reported by the studies were extracted. Only data available in the published studies and abstracts were used.

## Results

Initially, we selected 1663 studies after the literature search on the databases, and we identified and excluded 732 duplicates. Thus, 931 studies remained for further analysis.

**Figure 1:**
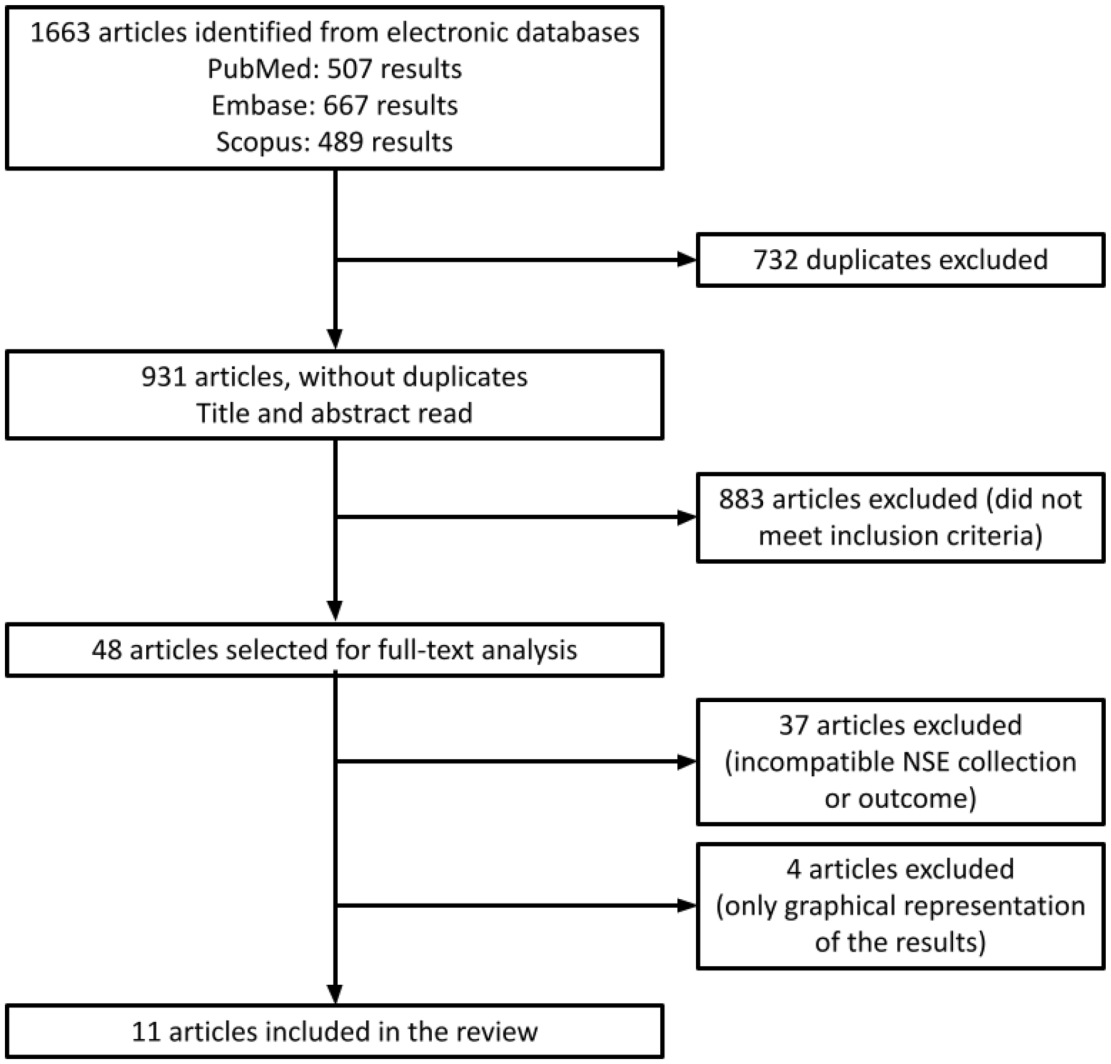
Diagram of study selection.

After the first phase of analysis (screening of title and abstract), 48 studies remained. Then, these studies were fully read by the investigators. In the second phase, 37 studies were excluded because they did not meet the inclusion criteria (no NSE collection at admission, no outcome of interest, or only a graphical representation of the results). The 11 selected studies constituted the systematic literature review (Fig. 1, Tables 1, 2).

**Table 1:** Key characteristics of the selected studies.

Author	Journal	Year	Country	N	Outcome(s)
Brea et al.^15^	Clin Chem Lab Med	2009	Spain	224	Functional outcome
Ghosh et al.^11^	Neuroimmunol and Neuroinflammation	2018	India	30	Stroke severity and Functional outcome
Jauch et al.^16^	Stroke	2006	USA	208	Stroke severity, Infarcted brain volume, and Functional outcome
Kurakina et al.^9^	Sovrem Tekhnologii Med	2021	Russia	50	Stroke severity, Infarcted brain volume, Functional outcome, and Death rates
Oh et al.^17^	Arch Neurolog	2003	South Korea	81	Stroke severity and Infarcted brain volume
Oh et al.^18^	Yonsei Med J	2002	South Korea	109	Stroke severity and Functional outcome
Park et al.^19^	Critical Care	2013	South Korea	175	Functional outcome
Purroy et al.^10^	Acta Neurol Scand	2021	Spain	332	Infarcted brain volume
Shash et al.^12^	The Egyptian J of Neurol, Psychiatry and Neurosurg	2021	Egypt	37	Stroke severity and Death rates
Singh et al.^20^	Clinica Chimica Acta	2013	India	100	Stroke severity
Wunderlich et al.^21^	Stroke	1999	Germany	52	Infarcted brain volume

**Table 2:** Outcome measurement tools and results of the selected studies (*Data presented as median [quartiles]; **Data presented as mean ± SD).

Author	Outcomes measurement	Results
Brea et al.^15^	Good (≤ 2) or bad (> 2) outcome, assessed by the mRS 90 days after the stroke onset	NSE levels at ED admission did not present statistical significance with the outcome (*p* = 0.067; good: 8.5 [5.2, 11.9]; bad: 9.2 [5.3, 12.1] ng/ml)*
Ghosh et al.^11^	Mild (0–4), moderate (5–15), or severe (16–42) stroke, assessed by the NIHSS at ED admission; mRS score < 2 or mRS score ≥ 2 30 days post-discharge	NSE levels at ED admission were positively correlated with the stroke severity (mild: 24.5 ± 5.4; moderate: 37 ± 11.9; severe: 56 ± 20.5 ng/ml) with statistical significance between mild and severe groups (*p* < 0.0001) and the functional outcome (mRS < 2: 30.6 ± 6.8; mRS ≥ 2: 47 ± 16.87 ng/ml) also with statistical significance (*p* = 0.0021)**
Jauch et al.^16^	NIHSS at ED admission; CT scans 24 h and three months after admission; mRS three months after admission	NSE levels were not associated with stroke severity, infarcted brain volume, or functional outcome, even after adjusting for treatment arm, history of hypertension, NIHSS at baseline, early ischemic changes on initial CT, and admission systolic blood pressure (*p* > 0.247)
Kurakina et al.^9^	NIHSS and GCS at ED admission; CT scan at ED admission; Good (≤ 2) or bad (> 2) outcome, assessed by the mRS 12–14 days after stroke onset; Death or not 14 days after the stroke onset	NSE levels at ED admission were positively correlated with the severity of neurological symptoms (r = 0.33; * p* = 0.02), the infarcted brain volume (r = 0.49; * p* = 0.003), the functional outcome (*p* = 0.04; good: 1.7 [1.4, 1.8]; bad: 2.1 [1.7, 3.0] ng/ml) and the death rates (*p* = 0.02; died: 3.0 [1.7, 6.0]; survived: 1.9 [1.5, 2.6] ng/ml) with statistical significance, but not with the GCS at ED admission (r = − 0.22; * p* = 0.15). The threshold of 2.6 ng/ml in NSE levels was established on the Receiver Operating Characteristic (ROC) curve to distinguish between patients who died and those who survived with a sensitivity of 74.7% and specificity of 71.4% (Area Under the Curve of 0.77; 95% Confidence Interval: 0.60–0.95; * p* = 0.02). NSE levels above 2.6 ng/ml during the acute ischemic stroke period were associated with an unfavorable prognosis regarding the likelihood of a lethal outcome (Odds Ratio = 8.3; * p* = 0.01)*
Oh et al.^17^	NIHSS at ED admission and MRI one week after the stroke onset	NSE levels at ED admission were positively correlated with the stroke severity (r = 0.42) and the infarcted brain volume (r = 0.62) with statistical significance (*p* < 0.002 and * p* < 0.001, respectively)
Oh et al.^18^	NIHSS at ED admission and mRS after three months of follow-up	NSE levels at ED admission were positively correlated with the stroke severity (r = 0.589) and the functional outcome (r = 0.635) with statistical significance (*p* < 0.05)
Park et al.^19^	Favorable (≤ 2) or poor (> 2) outcome, assessed by the mRS after three months of follow-up	NSE levels at ED admission did not correlate with the functional outcome (favorable: 5.6 [1.6, 10.3]; poor: 7.7 [3.0, 11.5]) with statistical significance (*p* = 0.14). Multivariate logistic regression analysis did not show statistical significance (*p* = 0.209), even after adjustment for age and initial NIHSS score (*p* = 0.325)*
Purroy et al.^10^	Diffusion-weighted imaging (DWI) within a week after the stroke onset	NSE levels at ED admission were significantly correlated with infarcted brain volume (*p* = 0.001; Spearman’s coefficient = 0.191). However, for a linear regression analysis adjusted by etiology, the correlation was lost
Shash et al.^12^	NIHSS at ED admission; Death or not 16 days after stroke onset	NSE levels at ED admission were significantly and positively correlated with clinical stroke severity among the patients (r = 0.737, * p* = 0.000), and this positive correlation was higher in the death group than in the no-death group (r = 0.853, * p* = 0.000 and r = 0.685, * p* = 0.03, respectively). The cutoff value of the NSE level to anticipate mortality at ED admission was > 33.45 ng/mL, with a sensitivity of 66.67% and specificity of 96.77%. The cutoff value of the NSE level to predict mortality at 48 h was > 31.04 ng/mL, with a sensitivity of 66.67% and specificity of 93.55%. The mean value of NSE level at ED admission was significantly higher in the death group (34.5 ± 6.2 ng/mL) than in the no-death group (23.1 ± 8.1 ng/mL) (*p* = 0.007)**
Singh et al.^20^	NIHSS at ED admission	Compared to the initial NSE levels in the mild (5.74 ± 1.16), moderate (12.05 ± 1.5), and severe groups (16.3 ± 0.58), NSE levels were found to be significantly higher in the severe group. NSE levels in stroke patients and the degree of neurological deficit were significantly correlated (r = 0.8, * p* ≤ 0.001)**
Wunderlich et al.^21^	CT scans within the first week after stroke onset	NSE levels at ED admission were significantly correlated with infarcted brain volume (r^2^ = 0.15, * p* = 0.005)

### Stroke severity versus NSE levels

Six studies showed a positive correlation between NSE levels and stroke severity, assessed by NIHSS at ED admission. Ghosh et al.^11^ found statistical significance between mild and severe stroke groups (*p* < 0.0001; mild: 24.5 ± 5.4 and severe: 56 ± 20.5 ng/ml). Kurakina et al.^9^, Oh et al.^17^, and Oh et al.^18^ associated higher NSE levels at ED admission with the severity of neurological symptoms (r = 0.33, *p* = 0.02; r = 0.42, *p* < 0.002; and r = 0.589, *p* < 0.05, respectively). Shash et al.^12^ also showed a positive correlation between NSE level and clinical stroke severity among the patients at ED admission (r = 0.737, *p* = 0.000). This correlation was higher in the death group than in the no-death group (r = 0.853, *p* = 0.000 and r = 0.685, *p* = 0.03, respectively).

Singh et al.^20^ compared the initial NSE levels in the mild (5.74 ± 1.16), moderate (12.05 ± 1.5), and severe groups (16.3 ± 0.58), and they found that the NSE levels and the degree of neurological deficits were significantly correlated (r = 0.8, *p* ≤ 0.001). In contrast, Jauch et al.^16^ showed that baseline NSE levels were not associated with stroke severity, even when considering multivariable models. Also, GCS at ED admission did not correlate with NSE levels (r = − 0.22, *p* = 0.15)^9^.

### Infarcted brain volume versus NSE levels

Four studies directly correlated infarcted brain volume to NSE levels. Kurakina et al.^9^ found a significant association using CT scans at ED admission (r = 0.49, *p* = 0.003). Oh et al.^17^ associated higher NSE levels with larger infarcted brain volume with MRI one week after the stroke onset (r = 0.62, *p* < 0.001). Purroy et al.^10^ and Wunderlich et al.^21^ correlated NSE levels with the size of stroke based on diffusion-weighted imaging (DWI) and CT scans, respectively, within a week of the stroke onset (*p* = 0.00, Spearman’s coefficient = 0.191 and *p* = 0.005, r^2^ = 0.15, respectively). Jauch et al.^16^ did not find a significant correlation between NSE levels and infarcted brain volume on 24-h and three-month post-stroke CT scans.

### Functional outcome versus NSE levels

Six studies analyzed the relationship between functional outcome and NSE levels. Ghosh et al.^11^ assessed the 30 days post-discharge outcome with mRS and found a statistical significance between the score and the NSE levels (*p* = 0.0021; mRS < 2: 30.6 ± 6.8; mRS ≥ 2: 47 ± 16.87 ng/ml). Kurakina et al.^9^ also associated NSE levels with the functional outcome (*p* = 0.04) and dichotomized into good (1.7 [1.4, 1.8]) and bad (2.1 [1.7, 3.0] ng/ml) outcomes, assessed by the mRS 12–14 days after stroke onset. The death rates 14 days after stroke onset were also presented with statistical significance (*p* = 0.02; died: 3.0 [1.7, 6.0]; survived: 1.9 [1.5, 2.6] ng/ml). The authors established that a cutoff point of 2.6 ng/ml in NSE levels was defined on the ROC curve to differentiate between patients who died and those who survived with a sensitivity of 74.7% and specificity of 71.4% (Area Under the Curve of 0.77; 95% Confidence Interval: 0.60–0.95; *p* = 0.02). Also, elevated NSE levels exceeding 2.6 ng/ml during the acute ischemic stroke period were linked to an unfavorable prognosis in terms of the likelihood of a lethal outcome (Odds Ratio = 8.3; *p* = 0.01).

Oh et al.^18^ also found a correlation between the functional outcome, assessed by the mRS after three months of follow-up, and the NSE levels with statistical significance (*p* < 0.05; r = 0.635). Shash et al.^12^ showed that the mean value of NSE level at ED admission was significantly higher in the 16 days after stroke onset in the death group (34.5 ± 6.2 ng/mL) than in the no-death group (23.1 ± 8.1 ng/mL) (*p* = 0.007). Also, they found that the cutoff value of the NSE level to anticipate mortality at ED admission was > 33.45 ng/mL, with a sensitivity of 66.67% and specificity of 96.77%. The cutoff value of the NSE level to predict mortality at 48 h was > 31.04 ng/mL, with a sensitivity of 66.67% and specificity of 93.55%.

Brea et al.^15^ found that NSE levels at ED admission were not statistically significant with the outcome assessed by the mRS 90 days after the stroke onset (*p* = 0.067; good: 8.5 [5.2, 11.9]; bad: 9.2 [5.3, 12.1] ng/ml). Similarly, Jauch et al.^16^ did not find a significant correlation with the functional outcome assessed by the mRS three months after admission (*p* > 0.247). Also, Park et al.^19^ dichotomized patients into favorable (≤ 2) and poor (> 2) outcomes based on the mRS score after three months of follow-up and did not present any correlation between the outcome and the NSE levels (*p* = 0.14; favorable: 5.6 [1.6, 10.3] and poor: 7.7 [3.0, 11.5]).

## Discussion

The present review analyzed the correlation between NSE levels at ED admission and the outcome of ischemic stroke patients by selecting 11 studies, from 1999 to 2021, with a total number of 1398 patients. Most studies (eight) reported a significant and positive correlation between the NSE levels and the stroke severity, the infarcted brain volume, or the functional outcome, in contrast to the other three studies.

Ischemic stroke is a global health concern, and the search for a reliable blood biomarker, such as troponin for ischemic heart disease or creatinine for renal failure, is ever-increasing^22–24^. Thus, NSE has emerged as a promising option since it is restricted to the brain tissue, and its elevation is already associated with some pathologies of the nervous system, such as traumatic brain injury and delirium^25,26^. Its clinical applicability is considered one among several other criteria (such as clinical deficits, validated scores, and imaging findings) to estimate the extent of brain injury^27,28^. However, NSE has not yet been established as part of routine clinical care.

In the present work, six studies found a significant correlation between NSE levels and stroke severity^9,11,12,17,18,20^. In contrast, only one did not present any association^16^. Four studies indicated a positive relationship between NSE levels and infarcted brain volume assessed by imaging^9,10,17,21^, while only one did not find an association^16^. For the functional outcome, four studies found a relationship^9,11,12,16^, while the other three studies did not achieve statistical significance in their findings^15,16,19^.

However, these data present several limitations and should be interpreted with caution. First, none of the studies provided conclusive evidence that just NSE can be used as a biomarker for predicting clinical outcomes. Second, the distribution of patients across the studies was highly heterogeneous. The three studies that did not find any correlation between NSE levels and stroke outcomes comprised a significant number of patients^15,16,19^.

Also, only three studies provided multivariate analyses or adjustments for other variables in their findings. Jauch et al.^16^ concluded that, after adjusting for the treatment arm, history of hypertension, baseline NIHSS, early ischemic changes on initial CT, and admission systolic blood pressure, the NSE levels remained not associated with the outcome (*p* > 0.247). Park et al.^19^ demonstrated that a multivariate logistic regression analysis of NSE between favorable and poor outcome groups did not show statistical significance (*p* = 0.209), even after adjusting for age and initial NIHSS score (*p* = 0.325). Purroy et al.^10^ concluded that the correlation between NSE levels at ED admission and infarcted brain volume was no longer significant after a linear regression analysis adjusted by etiology. Due to the lack of adequate statistical information to conduct a more rigorous analysis in most of the selected studies, we were unable to assess heterogeneity or perform a meta-analysis.

Another noteworthy limitation is the considerable variability in NSE levels observed across the selected studies. The mean values of NSE levels for stroke patients varied from 24.9 ± 8.8 to 0.0114 ng/mL^10,12^. Mild strokes ranged from 5.74 ± 1.16 to 24.5 ± 5.4 ng/mL, moderate from 12.05 ± 1.5 to 37 ± 11.9 ng/mL, and severe from 16.3 ± 0.58 to 56 ± 20.5 ng/mL^11,20^. Good outcomes (mRS ≤ 2) varied from 1.7 to 30.6 ± 6.8 ng/mL, while bad outcomes (mRS > 2) ranged from 2.1 to 47 ± 16.87 ng/mL^9,11^ when appropriate conversions to the ng/mL unit of measurement were made. The differences in the kits, techniques, and standard ranges used for assays might be another factor that contributed to this variation, which also highlights the challenge of standardizing NSE ranges.

Additionally, we excluded the studies in which NSE levels were collected within a few hours of admission or based on a specific time after the stroke onset to standardize the collection time (ED admission). However, the selected studies did not limit the maximum time between stroke onset and arrival at the healthcare facility for patient inclusion, allowing for considerable variations and, consequently, heterogeneity in the results.

In conclusion, the measurement of the NSE levels at ED admission can be a promising tool for predicting ischemic stroke patient prognostic but presents high discrepancies, lack of adjustment for comorbidities, age, and other confounding variables, and low robustness and applicability of the blood biomarker in clinical practice. This highlights the complex nature of using NSE as a standalone biomarker. Therefore, we suggest further studies employ a more precise definition of the collection time and greater standardization of reference values for the kits used in the laboratory assays, incorporating multivariate and more rigorous statistical analyses to establish and define the role of NSE in the prognosis of ischemic stroke patients.

## Data Availability

No datasets were generated or analyzed during the current study.
